# High-Speed Railway Intruding Object Image Generating with Generative Adversarial Networks

**DOI:** 10.3390/s19143075

**Published:** 2019-07-11

**Authors:** Baoqing Guo, Gan Geng, Liqiang Zhu, Hongmei Shi, Zujun Yu

**Affiliations:** 1 School of Mechanical, Electronic and Control Engineering, Beijing Jiaotong University, Beijing 100044, China; 2 Key Laboratory of Vehicle Advanced Manufacturing, Measuring and Control Technology, Ministry of Education, Beijing Jiaotong University, Beijing 100044, China

**Keywords:** railway intruding object, image generating, image translation, GAN

## Abstract

Foreign object intrusion is a great threat to high-speed railway safety operations. Accurate foreign object intrusion detection is particularly important. As a result of the lack of intruding foreign object samples during the operational period, artificially generated ones will greatly benefit the development of the detection methods. In this paper, we propose a novel method to generate railway intruding object images based on an improved conditional deep convolutional generative adversarial network (C-DCGAN). It consists of a generator and multi-scale discriminators. Loss function is also improved so as to generate samples with a high quality and authenticity. The generator is extracted in order to generate foreign object images from input semantic labels. We synthesize the generated objects to the railway scene. To make the generated objects more similar to real objects, on scale in different positions of a railway scene, a scale estimation algorithm based on the gauge constant is proposed. The experimental results on the railway intruding object dataset show that the proposed C-DCGAN model outperforms several state-of-the-art methods and achieves a higher quality (the pixel-wise accuracy, mean intersection-over-union (mIoU), and mean average precision (mAP) are 80.46%, 0.65, and 0.69, respectively) and diversity (the Fréchet-Inception Distance (FID) score is 26.87) of generated samples. The mIoU of the real-generated pedestrian pairs reaches 0.85, and indicates a higher scale of accuracy for the generated intruding objects in the railway scene.

## 1. Introduction

Foreign objects intruding railway clearance, such as pedestrians and large livestock, are a major hazard to the safety of railway operations. It is of great significance to detect intruding foreign objects quickly and accurately. Numerous intruding object samples are needed for detection algorithm development and testing. However, foreign object intrusion events are rare in daily operation. At the same time, experiments on operating high-speed railways are not permitted. Artificially generated railway images with intruding objects will benefit detection algorithm development and testing.

**Figure 1:**
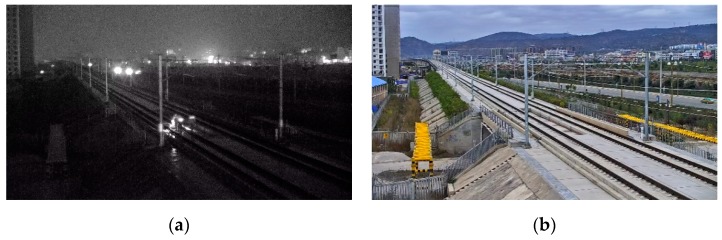
Comparison of images at non-operational and operational period: (**a**) railway intrusion sample at night; (**b**) images in operational period.

At present, railway foreign object intrusion detection methods mainly include contact type and non-contact type [1]. The contact detection method refers to the installation of a protective net along the railway in order to achieve the physical isolation of the railway boundary; non-contact methods include infrared, laser, and video surveillance. Video surveillance refers to the identification of foreign objects intruding the railway clearance using image processing. This method is widely used because of the advantages of being low cost, intuitive, and having a high accuracy. There are many algorithms for foreign objects intrusion detection. Teng Z [2] proposed a super-pixel-based railway foreign object intrusion detection algorithm, in which a support vector machine (SVM) was used to classify foreign objects and improve the detection accuracy. Tao Y [3] proposed an improved feature fusion convolutional neural network for foreign object intrusion detection in a railway shunting mode. It improved the detection efficiency and achieved a high accuracy through depthwise convolution. Yang Liuxu [4] proposed a railway foreign objects intrusion detection algorithm based on a fast background difference, which had a higher detection speed and was used for a demonstration application in the Shanghai–Nanjing high-speed railway of China. Wang Ning [5] proposed a railway intruding pedestrian classification algorithm based on an improved deep convolutional network, in which the improved AlexNet was combined with a HOG feature; the training and classification test on the railway intrusion foreign object datasets showed that it had a higher accuracy and real-time performance. All of the detection methods require large quantities of railway objects intruding as samples. The samples in the above algorithms were all obtained during a non-operational period at night. Figure 1a,b are the images of the same scene at non-operational and operational periods, respectively. The large gap makes it impossible to evaluate the existing detection methods during the operational period. A large amount railway foreign object intruding images during operational period are badly needed. But experiments for sample collection in operational period in daytime are not permitted. Therefore, it is of great significance to study the method of sample generating.

In recent years, the methods of machine learning have achieved great performance in many field [6,7,8,9,10]. In data generating, some physical models are available in some applications [11,12,13]. For images generated with machine learning, Goodfellow et al. [14] proposed generative adversarial networks (GAN) in 2014. GAN is derived from the Nash balance in game theory, and includes a generator (G) and a discriminator (D). The generator and discriminator have a confrontational relationship. They constantly optimize their parameters in the game in order to win and finally reach the Nash balance. In recent years, with the emergence of conditional GAN (CGAN) [15] and deep convolutional GAN (DCGAN) [16], GAN has gained widespread attention in the field of image generating. A variety of derived models have been proposed for different types of tasks or optimization methods. For example, Pix2pix [17], cycle-consistent adversarial networks (CycleGAN) [18], and other improved models [19,20] are proposed in order to solve the problem of image-to-image translation. Optimization methods such as Wasserstein GAN (W-GAN) [21] and least squares (LS)-GAN [22] have been proposed to solve problems of training instability and mode collapse. However, the GAN image generating method has the problem of low quality, and has not been used in the field of railway intruding object image generating.

In this paper, we propose a novel railway intruding object image generating method of high quality and authenticity, based on an improved conditional DCGAN (C-DCGAN), which consists of a generator and multi-scale discriminators. We also present the loss function so as to promote the quality and authenticity of the generated samples. For synthetizing the generated intruding objects to a railway scene with a high scale accuracy, the scale sizes of the generated objects in the different positions are calculated with the invariance of a gauge constant.

The major contributions include the following:

- A novel method for generating railway intruding object images is proposed based on an improved conditional DCGAN (C-DCGAN).
- In consideration of the authenticity and quality of the generated intruding objects, the generator, multi-scale discriminators, and novel loss function of the improved C-DCGAN model were constructed.
- An intruding-object scales estimation algorithm based on a gauge constant is presented so as to synthesize generated intruding objects to a railway scene with a high scale accuracy.
- A comprehensive evaluation strategy based on several metrics is proposed. With the experiments on the railway intruding object dataset, the proposed method outperforms several state-of-the-art methods and achieves a higher quality as well as diversity by metrics of pixel-wise accuracy, mean intersection-over-union (mIoU), mean average precision (mAP), and a Fréchet-Inception Distance (FID) score. The mIoU score of the generated–real pedestrian pairs reached 0.85, and shows the high-scale accuracy of the intruding objects in the railway scene.

The rest of this paper is organized as follows. Section 2 introduces the latest research and the related theories of GAN and image-to-image translation. The railway intruding object image synthesis method based on the C-DCGAN model and gauge constant is proposed in Section 3. Section 4 evaluates the authenticity and scale accuracy of the generated railway foreign objects by the experiments. Section 5 draws conclusions and discusses future research works.

## 2. Related Work

In this section, we cover the works of GAN, and discuss the latest developments of image-to-image translation.

### 2.1. Generative Adversarial Networks

**Figure 2:**
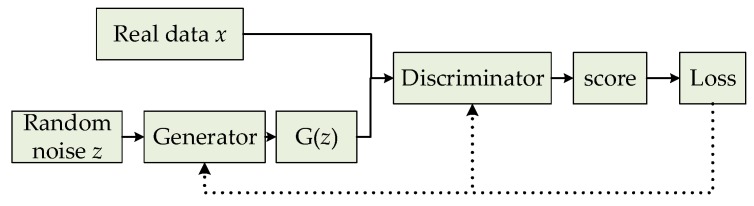
Basic framework of generative adversarial networks (GAN).

GAN usually includes a generator (G) and a discriminator (D), which are two independent neural networks. The generator takes a random noise (*z*) as the input. It learns the data distribution of the real samples and generates realistic fake samples that confuse the discriminator. The discriminator uses the real data (*x*) and the generated *G(z)* as an input to determine whether the input is a real sample (*x*) or a generated one. The basic framework of GAN is shown in Figure 2.

The implementation method of GAN is to make the generator and discriminator conduct confrontation training. The generator performs unsupervised learning without a large amount of prior knowledge in order to generate realistic data to confuse the discriminator. The discriminator cannot effectively distinguish whether the data is from real samples or generated ones. The generator and discriminator eventually reach the Nash balance. The objective function of GAN is shown as Equation (1).

(1) $$\underset{G}{\min}\;\underset{D}{\max}V\left(D,G\right)=E_{x\sim p_{data}(x)}[\log D(x)]+E_{z\sim p_{z}(z)}[\log\left(1-D\left(G\left(z\right)\right)\right)]$$

where, $x\sim P_{data}\left(x\right)$ represents a sample from the real data, $z\sim P_{z}\left(z\right)$ represents a generated sample, and *D(G(z))* represents the probability that the generated data is discriminated as a real sample.

**Figure 3:**
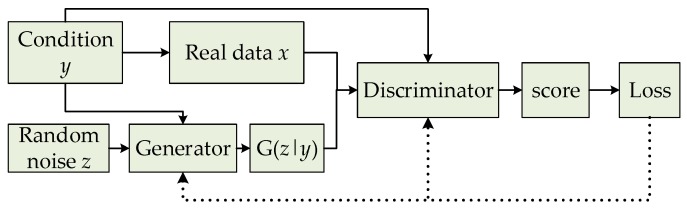
Basic framework of conditional GAN (CGAN).

However, this unsupervised learning without pre-modeling is too free. GAN has problems such as difficult training, model collapse, and a poor learning effect. In order to solve these problems, conditional GAN (CGAN) [15] is proposed so as to add a conditional variable (*y*) to both the generator and discriminator, as shown in Figure 3. Currently, the input noise (*z*) and conditional variable (*y*) form a joint hidden layer of representation information, and can be input into the generator for guiding data generating. Then, the optimization problem is transformed into a confrontational game with a conditional probability.

In an image processing task, convolutional neural networks (CNNs) [23] imitate the human visual perception mechanism, and use convolution operations to extract image features in order to achieve an excellent performance. Deep convolutional GAN (DCGAN) combines GAN with CNN by eliminating all of the pooling, using batch normalization (BN) and full convolutional structures, and changing the activation functions. Much progress has been made in the fields of image target detection [24], image dehazing [25], texture synthesis [26], and image translation [15].

In a GAN derivative model, the method proposed by Tobias Hinz [27] is closer to ours. The proposed model allows for the object to be added anywhere in the image by learning the objects in the bounding box. The SEIGAN [28] model is used for target segmentation and inpainting in the background images. However, it needs a complex dataset of object samples in different backgrounds for the model training.

In order to generate high-quality images, an optimization method of the model training is especially important. Aimed at gradient disappearance in the training process, Arjovsky proposed Wasserstein GAN (W-GAN) [21], which used Earth-Mover instead of Jensen-Shannon divergence as the criterion for measuring the distance between the real and generated samples. Least squares GAN (LS-GAN) [22] replaced the commonly used cross entropy loss function with the least squares loss to solve problems such as unstable training processes and low image quality.

### 2.2. Image-to-Image Translation Based on GAN

Image-to-image translation is a state-of-the-art method to generate intruding object images from the input semantic labels. Image-to-image is a derivative model based on CGAN, which changes the input to an image. Phillip Isola et al. proposed a general framework for image translation of Pix2pix [17]. The model translates the image from domain A to domain B with paired data training from both domains. It uses U-net [29] as a generator and PatchGAN [17] as a discriminator. The details of the generated image are improved obviously by a size of 256 × 256, but the quality of the generated higher-size image is poor. In order to break the limitation of paired data, CycleGAN [18], DiscoGAN [30], and DualGAN [31] models were proposed. CycleGAN is the most classic one, which contains two generators and two discriminators for separating the image content from the style through a loop-consistent mechanism. Only unpaired samples from both domains are needed in order to complete training. However, the quality of the generated images is worse than the Pix2pix framework. Because of the image blurring introduced by the alone use of L1 loss [32], the adversarial loss is added so as to enrich the image details in many studies [33,34]. However, the quality of these models for higher sizes is poor, and no reports show that they have been used in the field of railway foreign object generating.

## 3. Methodology

**Figure 4:**
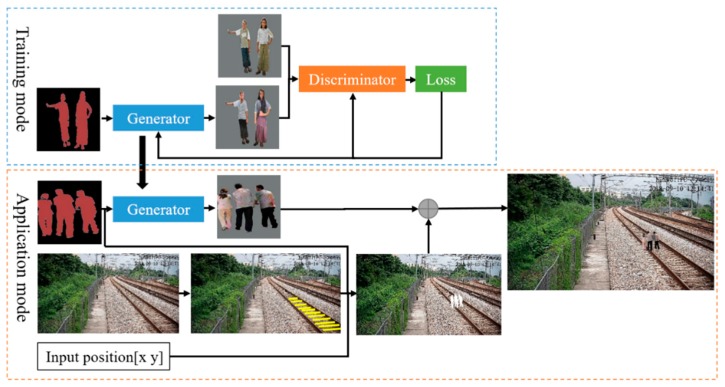
Overview of the railway intruding object image generating algorithm. The conditional deep convolutional GAN (C-DCGAN) model is first trained with image pairs of semantic labels and real images. For application, the trained generator is used to translate the semantic labels to various real images. The semantic image is also used to segment the objects’ contours in the railway scene. After the object scale size is calculated at the position, the generated intruding object is synthesized to the railway scene.

In order to generate high-quality and realistic railway intruding object images, we combine CGAN and DCGAN to construct a conditional DCGAN (C-DCGAN). The framework consists of the training mode and application mode, as shown in Figure 4. In the training mode, the C-DCGAN model is trained on the paired samples so as to learn the map from the semantic images to real images. In the application mode, the trained generator is then extracted to translate the input semantic image to a foreign object image in a higher size. At the same time, the scale of the generated foreign objects in different railway positions is calculated based on the invariance of the gauge constant. The foreign object is synthesized to the railway scene at pixel-level eventually.

### 3.1. C-DCGAN Model

The C-DCGAN model contains a generator and a discriminator, both of which are convolutional network structures for image feature information extracting.

**Figure 5:**
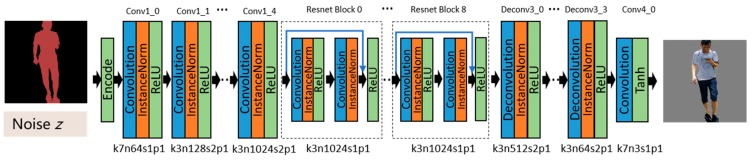
Architecture of the generator network with the corresponding kernel size (k), number of feature maps (n), stride (s), and padding (p) indicated for each layer. Convolution is used to extract the features. The features are transformed from the semantic domain to the real one in the ResNet blocks. Low-level features are restored with the deconvolution, and the real image is ultimately generated.

The generator adopts a full convolutional structure, consisting of five convolutional layers as encoders, nine residual modules (Resnet block) [35] as converters, and four deconvolutional layers as decoders. Table 1 shows the architecture of the generator. Firstly, the input semantic image preprocessed by one-hot encoding is input into the convolutional layers for encoding. The image is downsampled by the convolution of a stride of two, instead of pooling for reducing the loss of feature information. The convolutional layers extract the information from the feature maps and compress them into a 32 × 32 × 1024 tensor. ResNet blocks are introduced to convert the image features. Each residual module contains two convolutional layers, after which the feature map is directly added to the input through a shortcut connection so as to reduce the information loss during the conversion process. Meanwhile, the residual module can avoid the problems of degradation and gradient disappearance in such a deep network training. The tensor size is kept unchanged by the residual module layer. Then, the feature maps are upsampled by the deconvolutional layers and are restored to low-level feature maps. Finally, the maps are restored to an actual image. It should be noted that the ReLU activation function is used after each convolution layer, except the last one, to reduce the possibility of gradient disappearance and over-fitting. The last convolution layer uses a Tanh activation function. At the same time, in order to avoid gradient explosion and to speed up the convergence of the model, the instance normalization layer is added after each convolution layer [36]. The generator network is shown in Figure 5, where *k* means kernel size, *n* represents feature maps, *s* means stride, *d* means dilation, and *p* is padding.

**Figure 6:**
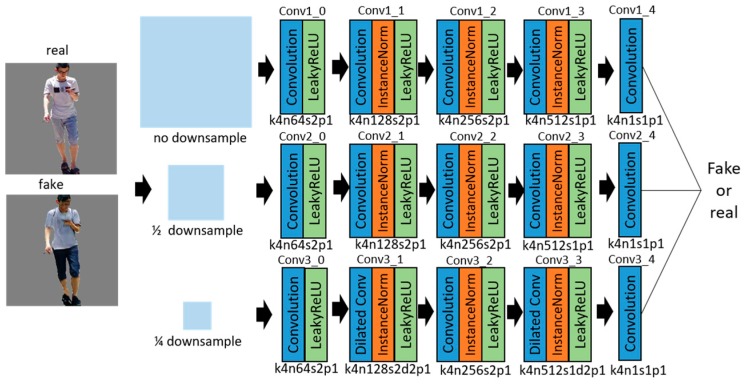
Multi-scale discriminators network with the corresponding kernel size (k), number of feature maps (n), stride (s), and padding (p) indicated for each layer.

The task of the discriminator is to discriminate between the real and generated samples at a higher size, under the consideration of the image global and local features. A deeper network or a larger convolution kernel can provide a larger receptive field for global features extracting, but there is the disadvantage of over-fitting. In this paper, the multi-scale discriminators network is used, which contains three discriminators models. They extract the features at original, 1/2, and 1/4 of the downsampled scales, as shown in Figure 6. The architecture of the multi-scale discriminators network is shown in Table 2. Each discriminator includes convolution, instance normalization, and LeakyReLU activation functions. The coarse-scale discriminator uses dilated convolution [37] instead of ordinary convolution to reduce the information loss and make the receptive field exponentially grow [38]. The fine scale discriminator focuses on the local detail information and guides the generator to produce finer images. The multi-scale discriminator network captures the image information to the greatest extent for higher-size image discrimination.

For the above multi-scale discriminators network, the GAN objective function is shown in Equation (2).

(2) $$\underset{G}{\min}\;\underset{D_{1},D_{2},D_{3}}{\max}\sum_{k=1,2,3}L_{GAN}\left(G,D_{k}\right)$$

where *k* is the index of the discriminator models.

In order to generate more realistic images, a feature matching loss [39] is introduced into the loss functions of each discriminator model. The feature maps of the generated and real images in each layer are matched with Equation (3).

(3) $$L_{FM}=E_{\left(s,x\right)}\sum_{i=1}^{T}\frac{1}{N_{i}}\left[{\left\|D_{k}^{i}\left(s,x\right)-D_{k}^{i}\left(s,G\left(s\right)\right)\right\|}_{1}\right]$$

where *T* is the index of the layers, $N_{i}$ represents the number of neurons in each layer, *s* represents the input semantic label, *x* stands for the real image sample, and *G*(*s*) is the generated image. The L1 distance constrained loss function is used to avoid the smooth blurring of the image caused by the L2 loss [40].

The perceptual loss [32] based on the pre-trained VGG16 model is added so as to guide clearer image generating. The loss function is defined as Equation (4).

(4) $$L_{VGG}=\sum_{i=1}^{N}\frac{1}{M_{i}}\left[{\left\|F^{i}\left(x\right)-F^{i}\left(G\left(s\right)\right)\right\|}_{1}\right]$$

where *i* is the corresponding index of layers in the VGG network, and *M_i_* denotes the elements number in layer *i*.

In order to make the training more stable and to improve the quality of the generated images, the least squares loss from LSGANS [22] is used. The final objective function is shown as Equation (5).

(5) $$\underset{G}{\min}\left(\left(\underset{D_{1},D_{2},D_{3}}{\max}\sum_{k=1,2,3}L_{GAN}\left(G,D_{k}\right)\right)+\lambda_{1}\sum_{k=1,2,3}L_{FM}\left(G,D_{k}\right)+\lambda_{2}L_{VGG}\right)$$

where *λ*_1_ and *λ*_2_ are weight of *L_FM_* and *L_VGG_*, respectively.

The training of the C-DCGAN model is an iterative process of the generators’ and discriminators’ optimizing. The training goal of the generator is to minimize the above objective function. The goal of the discriminator is to maximize the above function. In order to maintain the balance and prevent neither the discriminator nor generator from winning in the confrontation, the discriminator should be updated once after the generator, updating *k*(*k*>1) times in the training process.

### 3.2. Scale Estimation of Generated Intruding Object

**Figure 7:**
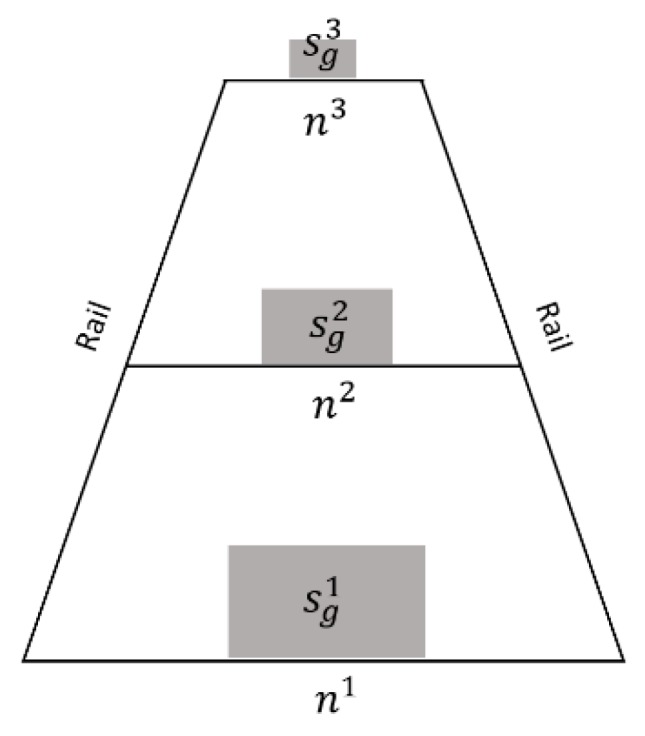
Scale size estimation based on the gauge constant. *n^1^, n^2^,* and *n^3^* represent the pixel numbers between two rails at different positions. With the invariant *s/g*, the pixel numbers of the generated objects at different positions of *s_g_^1^*, *s_g_^2^*, and *s_g_^3^* can be calculated when *n^1^, n^2^,* and *n^3^* are detected.

In order to synthesize the generated intruding object image to the railway scene with a higher scale accuracy, the ratio of the intruding object to gauge constant are used to estimate the pixel scale of the generated objects in different positions in the railway image, shown as Equation (6).

(6) $$\frac{s}{g}=\frac{s_{g}{}^{i}}{n^{i}}$$

where *s* is the real size of objects, *g* is the gauge constant (1435 mm), *s_g_^i^* represents the pixel number of the generated objects in the *i*th position, and *n^i^* is the pixel number between two rails in the *i*th position, as shown in Figure 7. For a certain category, *s/g*, *s_g_^i^*/*n^i^* are all constant. When the pixel numbers between rails *n^i^* are detected, the generated object pixel number (*s^i^_g_*) could be calculated at the same position.

**Figure 8:**
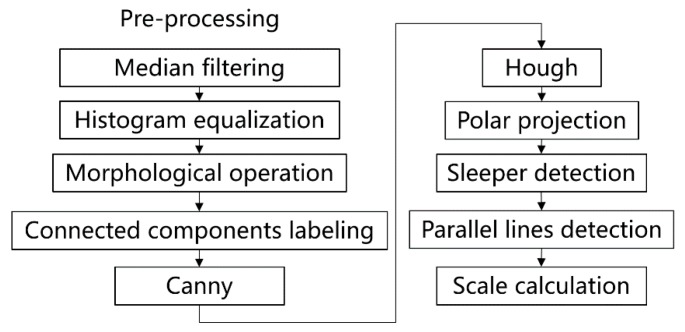
Overview of the generated object size estimation algorithm. The rail line features are highlighted with the pre-processing, including median filtering, histogram equalization, morphological operation, and connected components labeling. Then, the edges are extracted by the Canny. The rail lines are detected by Hough based on polar projection. Parallel sleepers are also detected for the scale size calculation.

An overview of the algorithm for detecting the pixel number between the rails at different positions is shown in Figure 8. Firstly, the rail lines are detected by the Hough transform after image pre-processing. Then, the Hough transform is used again to detect the sleeper lines between the rails. The pixel number between the two rails at a certain position can be obtained by the equation of the rails and sleeper lines. The pixel number of the generated objects can be calculated by Equation (6).

**Figure 9:**
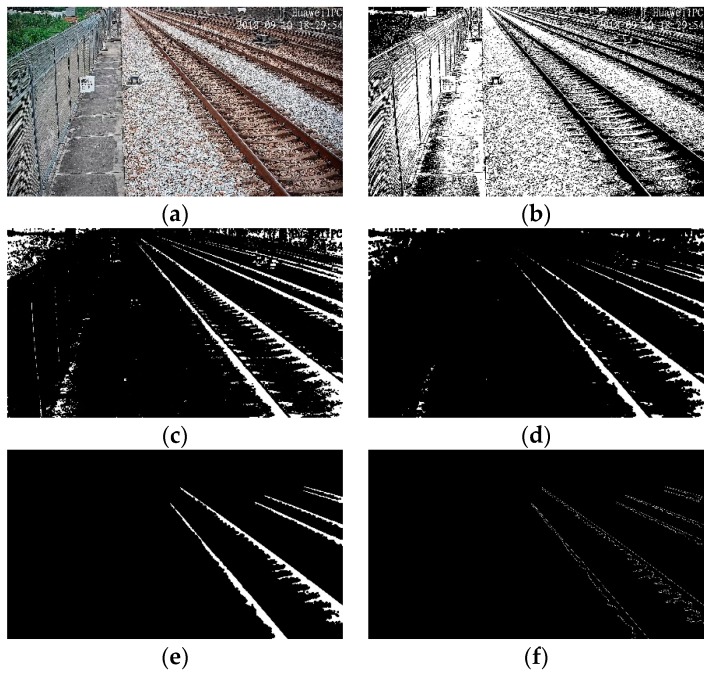
Railway scene image pre-processing: (**a**) original railway scene; (**b**) median filtered and binarization; (**c**) dilation; (**d**) erosion; (**e**) eight-connected components labeling; (**f**) Canny.

Because of the complicated railway scene and the many interference factors, it is necessary to pre-process the image in order to highlight the rail. Firstly, median filtering is used to filter the noises caused by vibration and other factors, and the rails after the larger threshold binarization and histogram equalization are further highlighted. In order to solve the problem of partial “fracture” caused by noise, the morphological close operation is used to the inverted image. The morphological close operation reconnects the “broken” part of the rail and eliminates most of the white spots caused by the ballasts, gravel, and plants, except for some independent white spots. They are eliminated with the eight-connected components labeling method. Then, the Canny edge detection operator is used to extract the edge of the rails for subsequent detection, as shown in Figure 9.

**Figure 10:**
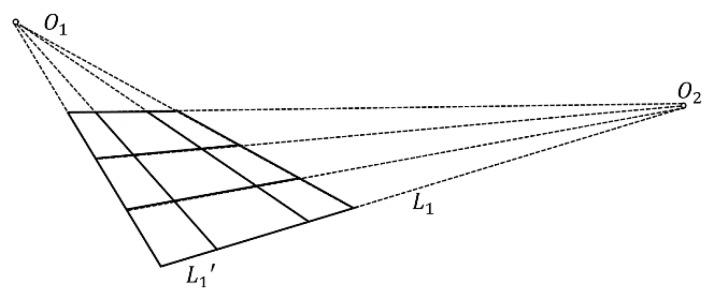
Vanishing point model for rails and sleepers [44].

**Figure 11:**
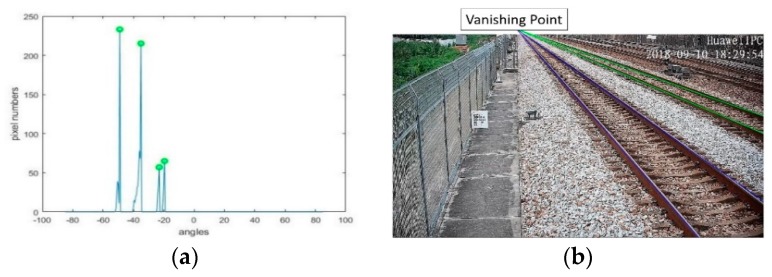
Parallel rails detection: (**a**) statistics of pixel numbers at different angles in polar projection; (**b**) rails detection.

After pre-processing, the rail features are outstanding, but it is still difficult to directly detect all of the rail lines. According to the perspective projective imaging model, parallel lines in the real world are mapped into lines intersecting at a point in the image plane, which is called the vanishing point. In a straight railway scene, all of the rails and sleepers are parallel to each other, respectively. The vanishing point model of the rails and sleepers is shown in Figure 10. The rails are intersected at point *O_1_*, and the sleepers are intersected at point *O_2_*. The Hough transform is a commonly used method for line detecting [41,42,43]. Here, we also used it to detect the two most significant straight rail lines and to determine their vanishing point *O_1_*. As all of the parallel rails pass through the vanishing point *O_1_*, the polar coordinate system can be established centered on the vanishing point. The polar projection method counts the white pixel numbers of the lines passing through the vanishing point in any direction. The peaks of the polar projection stand for the most obvious rails, as shown in Figure 11a. The parallel rails are detected in Figure 11b.

The sleeper area is segmented by the detected rails, and is pre-processed with the same steps for the rails. In the railway scene, the length of the sleeper lines between the rails is much smaller than the distance to the vanishing point. So, the lines of the sleeper can be considered approximately parallel. The Hough transform is used again to detect the sleeper lines between the rails. The detected lines are divided into 180 categories according to their slopes. The total length of the detected lines in each category can be calculated by the following:

(7) $$S_{i}=\sum_{j=1}^{N}l_{ij},(i=0,1,2,\ldots ,179)$$

where *N* is number of detected lines in each category, and *S_i_* denotes the total length of the detected lines (*l_ij_*) in the *i*th category.

**Figure 12:**
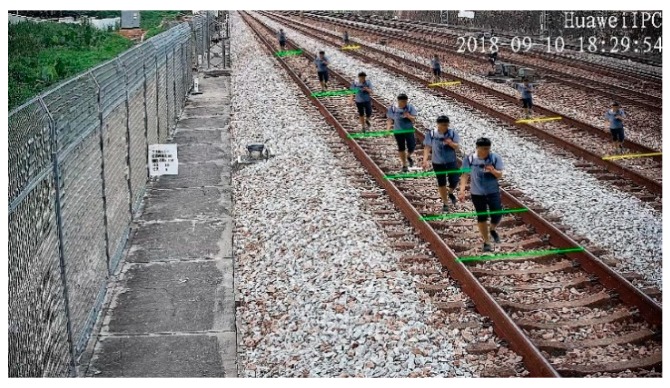
Scaled objects and sleeper lines at different positions.

The category with the largest *S_i_* is the angular direction of the parallel sleepers. The pixel number between the rails at different positions can be determined by the sleeper line segments. The pixel number of generated objects in the same position can be calculated in Equation (6), and the scaled objects and sleeper line segments are shown in Figure 12.

## 4. Experiments and Evaluations

In order to evaluate the authenticity, quality, and scale accuracy of the generated intruding object images in the railway scene, we established a railway intruding object dataset for image translation from semantic labels to real images, and a railway scene dataset as a background for image synthesis. We conducted experiments to evaluate the generated intruding foreign objects images with several metrics. Comparison results with other state-of-the-art methods (Pix2pix, CycleGAN, and DualGAN) and model optimizations are also provided.

### 4.1. Datasets and Training Details

Potential intruding objects on railways mainly include pedestrians and large livestock (sheep, horses, and cows). We first built a dataset of railway intruding object images derived from the public database. The MS-COCO dataset is one of the most commonly used datasets for deep learning, which includes 80-object categories and more than 200,000 labeled images [45]. The LIP dataset [46], containing images of 19 human body parts semantic labels, is one of the commonly used datasets in the field of pedestrian analysis. We built the dataset of railway intruding objects by the following steps:

(1) Semantic labels and real images of specified categories (pedestrian, sheep, cow, and horse) are extracted from the LIP and MS-COCO datasets.
(2) The extracted samples are resized to 512 × 512.
(3) According to the semantic labels, the objects are segmented from the background in the real images to reduce the influence of the complex background features on training.
(4) We reset the pixel values of each category in the semantic labels.

**Figure 13:**
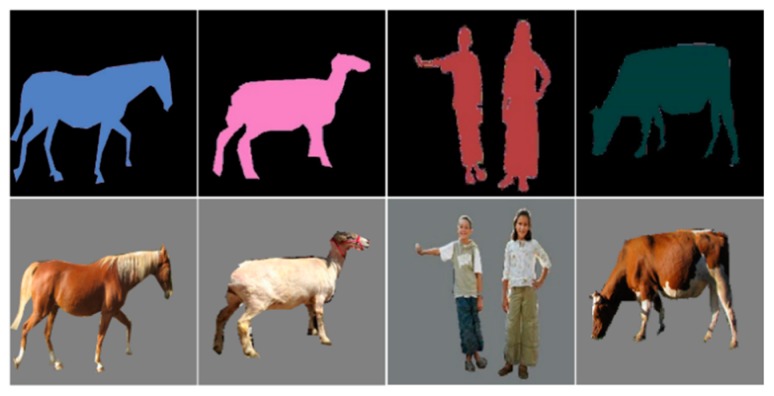
Samples of railway intruding object dataset.

Our dataset includes 11,615 semantic and real-image pairs of pedestrians, sheep, cows, and horses, as shown in Figure 13. The contents of the dataset are shown in Table 3. For the training set, we used 80% of random samples of each category. The remaining 20% of the samples we allocated to be the validation sets.

**Figure 14:**
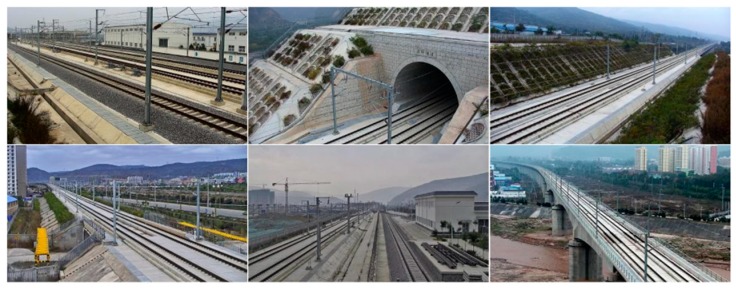
Samples of the railway scene dataset.

The railway scene dataset was constructed based on surveillance videos along the high-speed rail lines. The dataset contained different scenes, such as station throat areas, tunnel portals, railway main lines, and so on, under different weather conditions. The samples of the railway scene dataset are shown in Figure 14. The size of all of the samples is 1920 × 1080.

Our experiments were performed on an Intel(R) Core (TM) i7-6850CPU@3.2GHz processor, 16GB RAM, NVIDIA GeFore GTX Titan GPU, PyTorch deep learning framework. The parameters of the C-DCGAN model training are shown in Table 4. The training process was carried out for 300 iterations. The learning rate of the optimizer was 0.002, and linearly attenuated to 0 after 100 iterations. In order to maintain a counterbalance, the ratio (*k*) of the updating times of the discriminator to generator is 1:3. For avoiding the gradient disappearance during training, the instance normalization method [36] was used.

**Figure 15:**
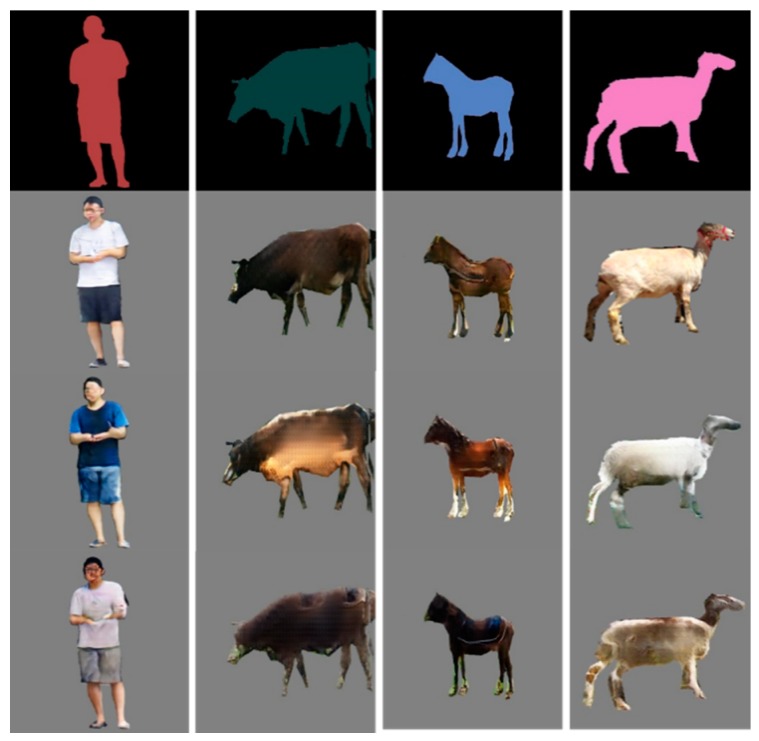
Generated intruding objects from the input semantic labels.

After the 96 h of training, the generator was extracted in order to generate intruding objects from the semantic labels. There were 8709 intruding objects of different categories that were generated. Some samples of diversity are shown in Figure 15. Every single sample generation took 327 ms.

### 4.2. Evaluation Metrics

For the railway intruding object image generating, we expected the generated samples to be of high quality, authenticity, and diversity. In order to comprehensively evaluate the generated samples, we employed four metrics.

To quantify the quality of the generated samples, we first adopted a similar evaluation protocol to previous works [18]. A popular semantic segmentation model, DeepLabv3+ [47], trained on our dataset, was used for semantic segmentation on the generated samples. Two standard semantic segmentation scores were used, including pixel-wise accuracy (Pixel acc) and mean IoU. They can be calculated by the comparison between the segmented label maps and the input ground truth label maps. The Pixel acc and mean IoU scores measure the interpretability and quality of the generated samples. The pre-trained DeelLabv3+ could obtain a close segmentation effect to that of the real samples on the realistic generated ones.

Diversified samples are of great significance to railway intruding detection methods. For the diversity assessment of the generated samples, we use the Fréchet-Inception Distance (FID) score [48], which indicates the distributions of inception embeddings (activations from the penultimate layer in the inception network) of the real and generated samples. A lower FID score shows a better diversity of generated samples.

We are also concerned about the overall authenticity of the generated railway intruding object image. So, the object detection network was used. Yolov3 [49] pre-trained with a MS-COCO dataset is a state-of-the-art object detection network with an abundant knowledge of different real objects in nature. It can be used as a judge to evaluate the authenticity and naturality of generated object images. The recall, precision, and AP score are employed in order to evaluate the authenticity. Specifically, the recall and precision could be calculated as Equation (8).

(8) $$\begin{array}{l} precision=TP/\left(TP+FP\right)\\ recall=TP/\left(TP+FN\right) \end{array}$$

where *TP*, *FP*, *TN*, and *FN* stand for true-positive, false-positive, true-negative, and false-negative, respectively. Under different confidence thresholds, the two-dimensional curve with precision and recall as the horizontal and vertical coordinates, respectively, can be plotted. The area under the curve is the average precision (AP), considering both the precision and recall. Usually, the higher the average precision is, the better the detection effect is. In our task, conversely, a higher average precision indicates a higher authenticity of the generated foreign objects.

**Figure 16:**
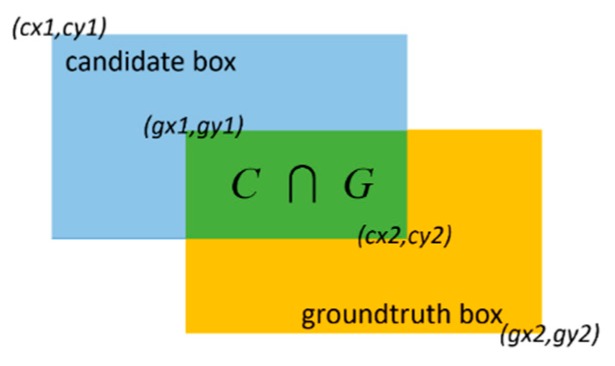
Intersection-over-union (IoU).

The scale accuracy of the generated intruding objects at different positions in the railway scene is essential to the authenticity of the synthesized samples. The intersection-over-union (IoU) is introduced in order to evaluate the scale accuracy of the generated objects. The IoU score refers to the overlap rate between the candidate and the groundtruth boxes, as shown in Figure 16 and Equation (9). The groundtruth and candidate boxes correspond to the real intruding objects and the generated ones at the same positions, respectively. In our task, a higher mean-IoU (mIoU) score indicates a higher scale accuracy of generated objects in a railway scene.

(9) $$IoU=\frac{area\left(C\right)\cap area\left(G\right)}{area\left(C\right)\cup area\left(G\right)}$$

### 4.3. Model Optimization

We optimized our C-DCGAN model based on the reference of previous works [17,18,22,29,50] and extensive experiments. As for the generator, with the loss functions and multi-scale discriminators fixed, we compared our generator with the following classical architectures: U-net [29] and CRN [50]. A case of six ResNet blocks was also tested. The semantic segmentation scores by each architecture are reported in Table 5. The highest scores of 80.458 show the best quality of generated samples by the nine-blocks generator. The 3 × 3 kernel size in the convolutional and deconvolutional layers and the building block of double 3 × 3 convolutions (instead of the bottleneck) of the proposed generator are proved in order to be better performers by comparison with other alternatives.

Multi-scale discriminators were compared with the conditions of one- or two-scale discriminators on our dataset. With the fixed nine-blocks generator and the full loss function, Table 6 shows the results, indicating that multi-scale discriminators improve the quality of the generated samples significantly. The dilated convolutions in the coarse scale improved the scores slightly.

We also studied the optimization of the loss functions. We added the feature matching the loss and VGG loss on the basis of GAN loss, respectively. The results of the different combinations on our dataset are shown in Table 7. It shows that the feature matching loss obviously improves the quality of generating, and that VGG loss enhanced the results slightly. Our final implementation achieved the best quality. Several combinations of weights (*λ*_1_, *λ*_2_) were tested, and the settings of 10 *λ*_1_ and 9 *λ*_2_ achieved the best results.

### 4.4. Evaluation and Comparison

Evaluations of the generated intruding objects and the synthesized samples were provided with the metrics mentioned above. Meanwhile, we compared the proposed method with state-of-the-art methods, Pix2pix [17], CycleGAN [18], and DualGAN [31], on our dataset.

**Figure 17:**
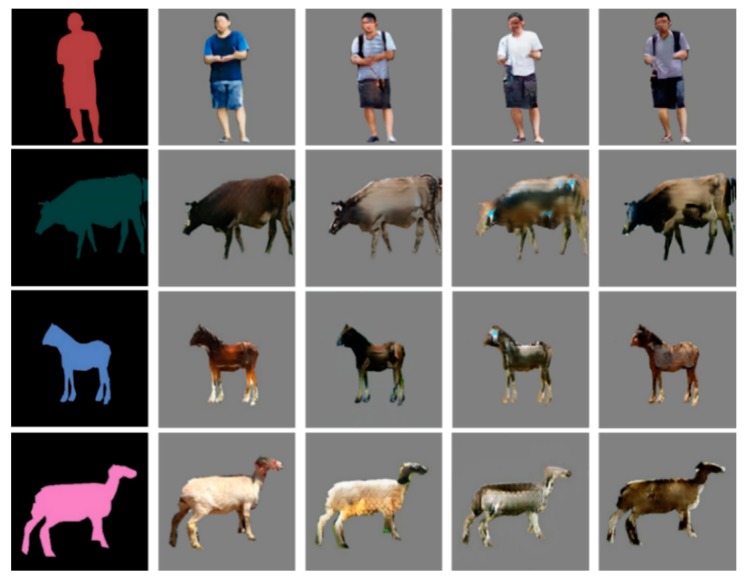
Generated samples: (from left to right) input semantic labels, and the generated samples by the C-DCGAN, Pix2pix, CycleGAN, and DualGAN, respectively.

With the same input semantic labels as ours, the generated samples by other methods are shown in Figure 17. Subjectively, the quality and diversity of the samples generated by our method are better than that of other methods. The semantic segmentation scores on the generated samples by different methods are reported in Table 8. The Pixel acc and mean IoU scores of our method are the highest, indicating that the samples generated by our method have a better quality than those by other methods on the pixel-level.

For the quantitative evaluation of diversity, the FID scores of different methods are listed in Table 9. The FID score of our method is 26.8, which is apparently lower than those of the other methods. The lowest FID score indicated that the samples generated by our method have the most diversity, which is of great significance to object-intruding detection.

**Figure 18:**
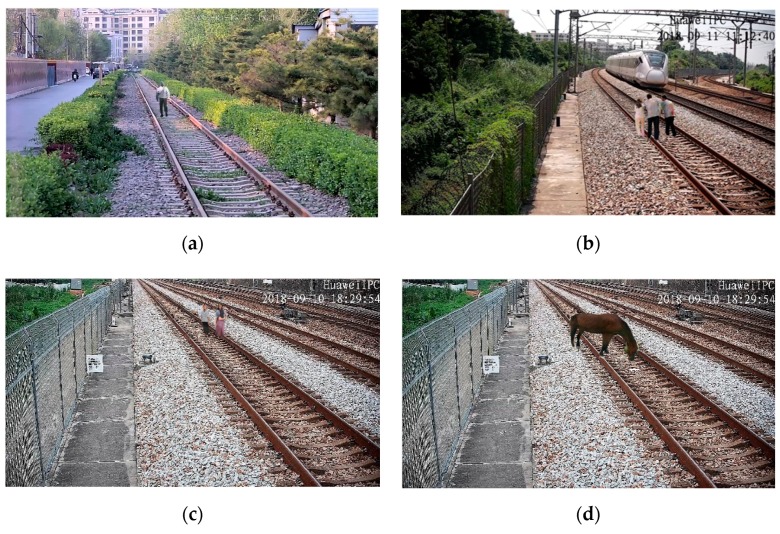
Samples of generated railway objects intruding on the image’s dataset. (**a**) A generated pedestrian in railway. (**b**) Three generated pedestrians on a railway. (**c**) Two generated pedestrians on a railway. (**d**) A generated horse on a railway.

**Figure 19:**
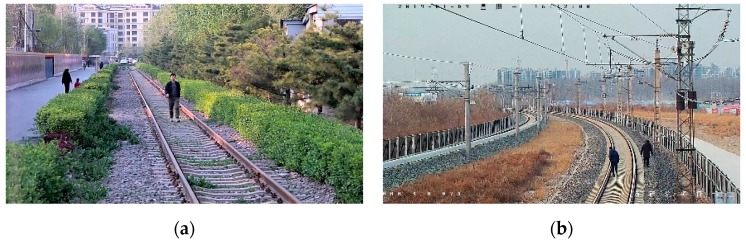
Samples of real railway objects intruding images dataset. (**a**) A real pedestrian on a railway. (**b**) Two real pedestrians on a railway.

**Figure 20:**
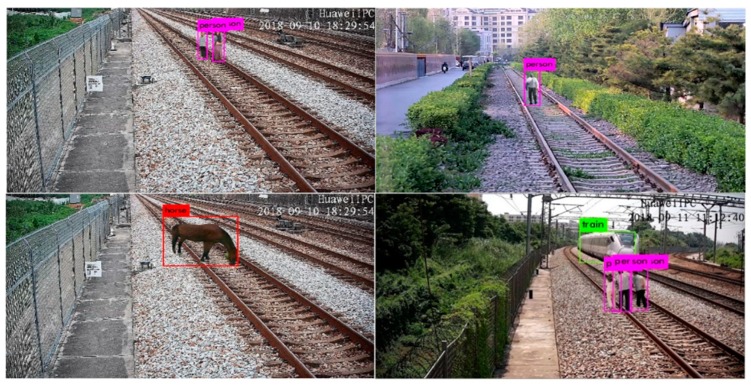
Detection results of generated intruding objects using our method.

With the method described in Section 3.2, 2529 railway intruding object images of different categories and positions, with our method, were synthesized as a generated railway object intruding images dataset, shown as Figure 18. As a contrast, a real railway object intruding images dataset was collected at a non-operational railway line, as shown in Figure 19. As a result of the limitation of the experimental conditions, only pedestrian intruding images were collected. The dataset includes 1265 images of pedestrians with a variety of postures and clothes colors. For evaluating the global authenticity of the synthesized images, both the generated and real railway object intruding images datasets were input into the pre-trained Yolov3 network, respectively. The average precision (AP) of each dataset was calculated, as shown in Table 10. In addition, in order to evaluate the authenticity of the generated images under a global coarse scale and local fine scale, the datasets were input into Yolov3 with different sizes. The detection results are shown in Figure 20.

As shown in Table 10, for pedestrians in a coarse scale of 320 × 320 input size, and 1198 generated intruding pedestrian images, the AP is 0.578, which is close to the 0.534 of the 1265 real ones. At finer sizes of 416 × 416 and 608 × 608, the AP of the generated intruding pedestrian images were 0.691 and 0.847, respectively. The AP of the real ones were 0.656 and 0.823. The little gap of AP between the two datasets indicates the authenticity of the generated pedestrians by our method. As a result of the lack of contrasting real livestock samples, only the AP of the generated ones were calculated. For the horses, cows, and sheep, their APs were higher than that of the pedestrians at different input sizes, respectively. The reason is that they are realistic and usually bigger than pedestrians. The experiment results show that our method could generate railway object intruding images with a high authenticity. The confusion matrices are shown in Table 11, with the 0.5 confidence threshold and 0.5 IoU threshold of the pre-trained Yolov3 model. The values on the horizontal ordinate are the category prediction results and the missed ones. The vertical axis shows the true categories. The higher values on the diagonal indicate the naturalness and authenticity of the generated samples by our method.

There were 2529 generated samples by other methods that were also synthesized to the same railway scene. The synthesized railway intruding object images were feed to the pre-trained Yolov3 with size of 416 × 416. The AP scores of the different methods are reported in Table 12, for quantitative evaluation. The scores of Pix2pix and our method are obviously higher than those of CycleGAN and DualGAN. It indicates that models of supervised learning such as Pix2pix and ours have a better performance than the unsupervised ones. Our method produced the highest mAP of 0.685, which is much better than any of the other models, indicating that our method is superior to the other three models on our dataset.

**Figure 21:**
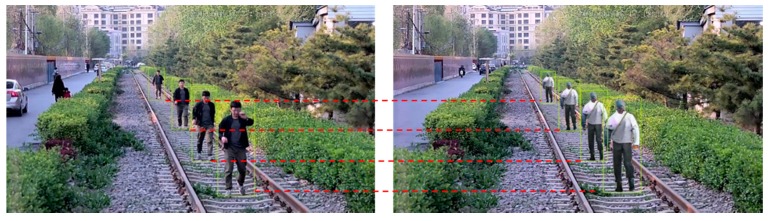
Scale evaluation of real and generated pedestrians. (**Left**) Real pedestrians (groundtruth boxes) and (**right**) generated pedestrians (candidate boxes) at corresponding positions.

In order to evaluate the scale accuracy of the generated objects, a pedestrian walked along the rail from far to near. The pedestrians are annotated as groundtruth boxes at different positions, as shown on the left in Figure 21. The generated pedestrians were synthesized to the railway scene at corresponding positions to the candidate boxes, as shown on the right in Figure 21. The corresponding groundtruth and candidate boxes were considered as a pedestrian pair. The mIoU scores of the single, double, and multiple pedestrians at different positions are shown in Table 13.

In Table 13, For a single pedestrian at a close, middle, and far distance in a railway scene, a real pedestrian and a generated one at a corresponding position were considered as a pair. The IoU score was used to evaluate the scale overlap between them. The IoU scores of 600 pedestrian pairs in different distances were calculated. With the increase of distance, the mIoU decreases. The lowest (0.821) in the far distance is still at a high level, indicating the scale size accuracy of the generated pedestrians. In cases of double and multiple pedestrians, the mIoU scores of the different distances also remained at a high level. The total mIoU (0.854) indicates that the generated pedestrians have a similar scale size to the real ones at different corresponding positions, which ensures the authenticity of the synthesized samples.

## 5. Conclusions

In this paper, a novel method for generating railway intruding object images of a high quality and authenticity is proposed. The method is based on an improved conditional DCGAN (C-DCGAN), which consists of a generator and multi-scale discriminators. For synthetizing the generated intruding objects to a railway scene with a high scale accuracy, an intruding objects scales estimation algorithm based on the gauge constant is also presented. The experimental results on the railway intruding object dataset show that the generated railway intruding object images are of a high quality, diversity, and scale accuracy, and they can be used for the training and testing of the intruding detection algorithm.

However, there are still some limitations for our method. The proposed method could only generate limited categories of intruding objects. Meanwhile, the quality of the generated image could be further improved.

In future works, we plan to enrich our railway intruding object dataset with more categories, such as running, climbing guardrail, and so on. We will develop a test platform for railway intruding object detection algorithms based on our method. Furthermore, we want to try the Hough and polar projection methods in applications of road-following and traffic analysis.

## Figures and Tables

**Table 1:** Architecture of the generator.

Layer_Name	Input_Size	Filters	Kernel_Size	Stride	Output_Size	Others
ReflectionPad0	512 × 512	-	-	-	518 × 518	-
Conv1_0	518 × 518	64	7 × 7	1.1	512 × 512	-
Conv1_1	512 × 512	128	3 × 3	2.2	256 × 256	-
Conv1_2	256 × 256	256	3 × 3	2.2	128 × 128	-
Conv1_3	128 × 128	512	3 × 3	2.2	64 × 64	-
Conv1_4	64 × 64	1024	3 × 3	2.2	32 × 32	-
Conv	32 × 32	1024	3 × 3	1.1	32 × 32	ResNet blocks × 9
Conv	32 × 32	1024	3 × 3	1.1	32 × 32	
Shortcuts	32 × 32	1024	3 × 3	1.1	32 × 32	
Deconv3_0	32 × 32	512	3 × 3	2.2	64 × 64	-
Deconv3_1	64 × 64	256	3 × 3	2.2	128 × 128	-
Deconv3_2	128 × 128	128	3 × 3	2.2	256 × 256	-
Deconv3_3	256 × 256	64	3 × 3	2.2	512 × 512	-
ReflectionPad1	512 × 512	-	-	-	518 × 518	-
Conv4	518 × 518	3	7 × 7	1.1	512 × 512	-

**Table 2:** Architecture of the multi-scale discriminators.

Module	Layers	Input_Size	Filters	Kernel_Size	Dilation	Stride	Output_Size
D1	Conv1_0	512 × 512	64	4 × 4	1	2.2	256 × 256
	Conv1_1	256 × 256	128	4 × 4	1	2.2	128 × 128
	Conv1_2	128 × 128	256	4 × 4	1	2.2	64 × 64
	Conv1_3	64 × 64	512	4 × 4	1	1,1	63 × 63
	Conv1_4	63 × 63	1	4 × 4	1	1.1	62 × 62
D2	Conv2_0	256 × 256	64	4 × 4	1	2.2	128 × 128
	Conv2_1	128 × 128	128	4 × 4	1	2.2	64 × 64
	Conv2_2	64 × 64	256	4 × 4	1	2.2	32 × 32
	Conv2_3	32 × 32	512	4 × 4	1	1.1	31 × 31
	Conv2_4	31 × 31	1	4 × 4	1	1.1	30 × 30
D3	Conv3_0	128 × 128	64	4 × 4	1	2.2	64 × 64
	Conv3_1	64 × 64	128	4 × 4	2	2.2	32 × 32
	Conv3_2	32 × 32	256	4 × 4	1	2.2	16 × 16
	Conv3_3	16 × 16	512	4 × 4	2	1.1	15 × 15
	Conv3_4	15 × 15	1	4 × 4	1	1.1	14 × 14

**Table 3:** Dataset of railway intrusion objects.

Categories	Number	Size (Pixels)
Pedestrian	4897	512 × 512
Sheep	1594	512 × 512
Cow	2055	512 × 512
Horse	3069	512 × 512

**Table 4:** Parameters of conditional deep convolutional generative adversarial networks (C-DCGAN) model training.

Size	Batch Size	*λ_1_*	*λ_2_*	*k*	Optimizer	Learning Rate	Momentum
512 × 512	1	10	9	1:3	Adam	0.0002	0.5

**Table 5:** Semantic segmentation scores of different generators. IoU—intersection-over-union.

Architectures	Pixel Acc (%)	Mean IoU
U-net	74.094	0.403
CRN	68.259	0.428
Ours (6 blocks)	76.549	0.547
Ours (9 blocks)	80.458	0.651

**Table 6:** Semantic segmentation scores of different discriminators.

Architectures	Pixel Acc (%)	Mean IoU
Single D	72.142	0.504
Double Ds	76.981	0.591
Triple Ds (without dilated conv)	79.452	0.640
Ours (with dilated conv)	80.458	0.651

**Table 7:** Semantic segmentation scores of different losses.

Architectures	Pixel Acc (%)	Mean IoU
Only GAN loss	70.843	0.457
GAN loss + feature matching loss	77.824	0.602
GAN loss+ VGG loss	72.176	0.483
Ours	80.458	0.651

**Table 8:** Semantic segmentation scores of generated samples by different methods.

	Pix2pix	CycleGAN	DualGAN	Ours	Real Samples
Pixel acc (%)	72.653	63.441	63.885	80.458	85.782
Mean IoU	0.441	0.347	0.358	0.651	0.724

**Table 9:** Fréchet-Inception Distance (FID) scores of the different methods.

	Pix2pix	CycleGAN	DualGAN	Ours	Real Samples
FID	45.42	47.13	48.62	26.87	13.59

**Table 10:** Detection results of generated and real datasets. AP—average precision.

Categories	Input Size	Datasets	Amount	AP
Pedestrian	320	Real	1265	0.534
		Generated	1198	0.578
	416	Real	1265	0.656
		Generated	1198	0.691
	608	Real	1265	0.823
		Generated	1198	0.847
Horse	320	Generated	447	0.625
	416	Generated		0.721
	608	Generated		0.829
Cow	320	Generated	472	0.611
	416	Generated		0.695
	608	Generated		0.844
Sheep	320	Generated	412	0.592
	416	Generated		0.631
	608	Generated		0.818

**Table 11:** Confusion matrices of generated samples using Yolov3.

		Prediction			
		Pedestrian	Horse	Cow	Sheep
**True labels**	**Pedestrian**	868	48	61	68
	**Horse**	20	319	38	25
	**Cow**	25	29	335	19
	**Sheep**	24	20	15	328

**Table 12:** AP scores of the generated samples using a different method.

Models	AP-Pedestrian	AP-Sheep	AP-Cow	AP-Horse	mAP
Pix2pix	0.625	0.558	0.593	0.627	0.601
CycleGan	0.501	0.498	0.519	0.526	0.511
DualGan	0.516	0.511	0.508	0.522	0.514
Ours	0.691	0.631	0.695	0.721	0.685

**Table 13:** IoU scores of pedestrian pairs.

Pedestrians	Position	Pair numbers	mIoU
Single	Far	198	0.889
	Middle	213	0.875
	Close	189	0.821
Double	Far	197	0.891
	Middle	230	0.862
	Close	196	0.812
Multiple	Far	195	0.857
	Middle	205	0.861
	Close	214	0.814
Total	—	1837	0.854
